# A new strategy to express the extracellular α-amylase from *Pyrococcus furiosus* in *Bacillus amyloliquefaciens*

**DOI:** 10.1038/srep22229

**Published:** 2016-02-26

**Authors:** Ping Wang, Peili Wang, Jian Tian, Xiaoxia Yu, Meihui Chang, Xiaoyu Chu, Ningfeng Wu

**Affiliations:** 1 Biotechnology Research Institute, Chinese Academy of Agricultural Sciences, Beijing 100081, China

## Abstract

Extracellular α-amylase from *Pyrococcus furiosus* (PFA) shows great starch-processing potential for industrial application due to its thermostability, long half-life and optimal activity at low pH; however, it is difficult to produce in large quantities. In contrast, α-amylase from *Bacillus amyloliquefaciens* (BAA) can be produced in larger quantities, but shows lower stability at high temperatures and low pH. Here, we describe a BAA protein expression pattern-mimicking strategy to express PFA in *B*. *amyloliquefaciens* using the expression and secretion elements of BAA, including the codon usage bias and mRNA structure of gene, promoter, signal peptide, host and cultivation conditions. This design was assessed to be successful by comparing the various genes (*mpfa* and *opfa*), promoters (PamyA and P43), and strains (F30, F31, F32 and F30-∆amyA). The final production of PFA yielded 2714 U/mL, about 3000- and 14-fold that reportedly produced in *B*. *subtilis* or *E*. *coli*, respectively. The recombinant PFA was optimally active at ~100 °C and pH 5 and did not require Ca^2+^ for activity or thermostability, and >80% of the enzyme activity was retained after treatment at 100 °C for 4 h.

Thermostable α-amylase is an important commercial enzyme, with a wide range of applications in starch processing, brewing, alcohol production, baking, textile production, paper recycling and other industries^1^. Since 1980, the most widely used enzyme for processing starch has been α-amylase from *Bacillus licheniformis* (BLA)^2^,^3^, a thermostable enzyme that functions optimally at 90 °C and pH 6–7 and requires the addition of calcium (Ca^2+^) for its thermostability^4^. However, these conditions are substantially different from those encountered in starch liquefaction, a process in which this enzyme is widely utilised. For example, the dissolving starch fraction requires a pH of ~4.5, and the next processing step involves liquefaction of starch to glucose oligomers, ideally performed at pH 4.5 and 105 °C; however, BLA is unstable under these conditions. Therefore, the pH must be adjusted to ~6.0 and Ca^2+^ added, thereby increasing the number of industrial processing steps and cost^5^. Furthermore, thermostability of α-amylase is an important property for its industrial use. The starch liquefaction process occurs at 95–105 °C^6^, whereas the half-life of residual BLA enzymatic activity is only 20 min at 95 °C.

The disparity between industrial requirements and the optimal activity of BLA has prompted research in two areas: (i) optimising the operative pH range, amount of Ca^2+^ salt added, and the thermostability of BLA^7^,^8^,^9^,^10^ or other α-amylases^11^; and (ii) investigating new thermostable α-amylases from hyperthermophilic bacteria and archaea^12^. Among these, extracellular α-amylase from *Pyrococcus furiosus* (PFA) functions optimally at 98 °C and retains >80% of maximal activity at pH 4.5 without the addition of Ca^2+^ salt^13^. Moreover, the half-life of PFA is >12 h at 100 °C without Ca^2+^ salt addition, while that of BLA is 20 min at 95 °C with 1 mM CaCl_2_^14^; Furthermore, PFA has nearly twice the activity at 98 °C as BLA has at 90 °C^15^. Such properties make PFA a potentially useful candidate for industrial application.

However, due to the inherent difficulty in cultivating *P*. *furiosus*, obtaining a sufficient number of cells for large-scale enzyme production has been problematic^16^. Several studies have attempted to express large quantities of PFA in *Escherichia coli*^13^,^15^,^16^,^17^,^18^, *Bacillus subtilis*^18^ and yeast^18^,^19^. However, production quantities were low, yielding ~195 U/mL in *E*. *coli*^17^, 0.7 U/mL in *B*. *subtilis*^18^ and 220 U/mL in yeast^19^. Higher production levels resulted in the accumulation of insoluble PFA, in the form of inclusion bodies^15^,^16^,^17^. Several studies have attempted to diminish inclusion body formation during PFA expression^15^,^17^, but it has resulted in lower levels of total recombinant protein production. Glycerol extraction methods to purify recombinant PFA from the inclusion bodies have been described^16^,^20^; however, this approach is impractical for industrial application.

*B*. *subtilis* and its close relatives are widely used in the pharmaceutical, agricultural and food industries. *B*. *subtilis*, *B*. *amyloliquefaciens* and *B*. *licheniformis* account for ~50% of the world’s industrial enzyme production, including proteases, α-amylases, β-glucanases, and penicillin acylases^21^,^22^. Among these strains, *B*. *amyloliquefaciens* displays a strong secretory ability but produces only a small amount of its own secretory protein, which is distinct from *B*. *subtilis*. In addition, *B*. *amyloliquefaciens* displays a greater growth rate with larger cell density than *B*. *licheniformis*. These data indicate that *B*. *amyloliquefaciens* is an ideal host for the secretory production of foreign proteins. Besides, up to 10–30 mg/mL high yield of BAA (α-amylase from *Bacillus amyloliquefaciens*) can be produced by *B*. *amyloliquefaciens*. Since BAA and PFA are α-amylases with similar three-dimensional structures^14^, could the expression pattern of BAA provide a reference for producing PFA? A recent study^23^ showed that a DNA methylation pattern-mimicking pipeline, mimicking the DNA methylation pattern of host to avoid the degradation of host, could overcome the restriction barrier of bacteria. Similarly, we designed a BAA protein expression pattern-mimicking strategy to overcome the barrier of secretory expression of PFA by competitively using the expression and secretion machines of BAA. The “mimicry” includes the codon usage bias and mRNA structure, promoter, signal peptide, expression vector, host and cultivation conditions of BAA. Meanwhile, the multiply-copied vector would provide PFA an advantage in competitive expression and secretion of BAA.

## Results

### Optimisation of transformation conditions

The electroporation conditions to transform recalcitrant *B*. *amyloliquefaciens* were optimised. The transformation efficiency was extremely low and no positive clones were detected when using the plasmids extracted from Top10, DH5α, Mach1, JM109 or JM110. In addition, heat inactivation of the host restriction modification systems^24^ showed no significant effects on the transformation efficiency. However, transformation using plasmids extracted from B19 (which expresses *B*. *amyloliquefaciens* methyltransferases) resulted in a transformation efficiency of at least 2.1 × 10^2^ cfu/μg plasmid DNA in the *B*. *amyloliquefaciens* strains (Table 1).

### BAA protein expression pattern-mimicking

**Figure 1:**
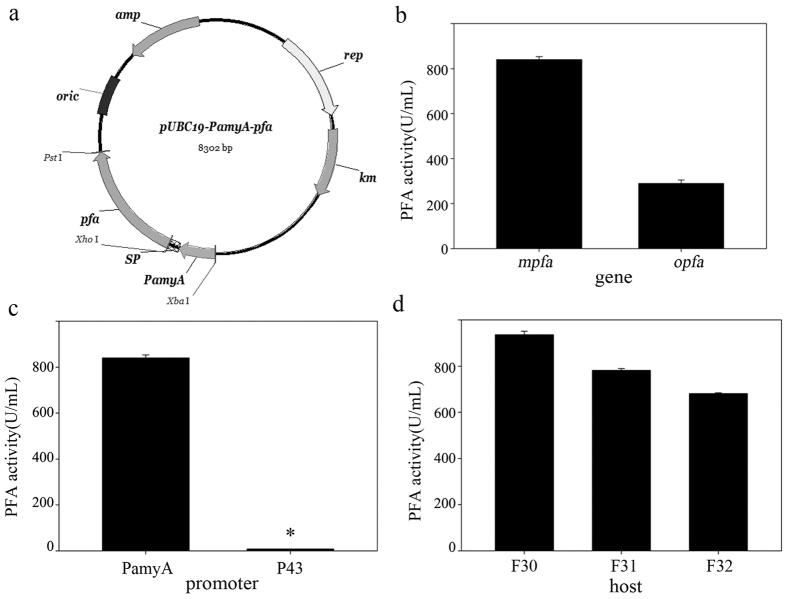
Expression of α-amylase from *Pyrococcus furiosus* (PFA) in *Bacillus amyloliquefaciens* using a BAA protein expression pattern-mimicking strategy. PFA activity of transformants in culture supernatants was determined using standard enzyme assays after 72 h of cultivation in fermentation medium. (**a**) Schematic representation of plasmid pUBC19-PamyA-*pfa*; (**b**) gene optimisation (pUBC19-PamyA-X). X represents *mpfa* or *opfa*; (**c**) promoter optimisation (pUBC19-X-*mpfa*). X represents PamyA or P43; (**d**) host optimisation. Three *B*. *amyloliquefaciens* strains, F30, F31 and F32, were chosen. Enzyme activity is expressed as the mean of three samples, and error bars indicate standard deviations (SD). *Unstable.

The BAA protein expression pattern-mimicking strategy was designed to include the promoter (PamyA) and signal peptide (SP) of *amyA* (the BAA gene of *B*. *amyloliquefaciens* F30). The target genes were two synthetic *pfa* genes (*mpfa* and *opfa*) that were designed based on the coding sequence of the wild-type PFA; *mpfa* was designed to mimic the mRNA structure and codon usage of *amyA* using the Biosoft VGD, and *opfa* was optimised and synthesised by the company GenScript using their proprietary algorithms (see “supplementary information”, Figure S1). No changes in the amino acid sequence of PFA were introduced. Finally, the plasmids pUBC19-PamyA-*mpfa* and pUBC19-PamyA-*opfa* were constructed (Fig. 1a). As shown in Fig. 1b, replacing *mpfa* with *opfa* resulted in a ~3-fold decrease in PFA activity. To assess the effect of the promoter, the starch-induced promoter PamyA was replaced with the constitutive promoters P43^25^, generating the vector pUBC19-P43-*mpfa*. As shown in Fig. 1c, the replacement resulted in a drastic decrease in PFA activity and little residual PFA activity. Subsequently, we found that the pUBC19-PamyA-*mpfa* plasmid was stable in F30, but pUBC19-P43-*mpfa* were not. Two additional *B*. *amyloliquefaciens* strains (F31 and F32) that produced α-amylases were used as hosts. As shown in Fig. 1d, PFA-F30 demonstrated the highest PFA activity, with ~20% and 40% greater activity than that expressed by PFA-F31 and PFA-F32, respectively.

These data demonstrate that the strategy was successful for PFA. The PFA-F30 strain (F30 harbouring the plasmid pUBC19-PamyA-*mpfa*) was chosen to carry out the subsequent experiments.

### Expression of recombinant PFA in *B*. *amyloliquefaciens*

**Figure 2:**
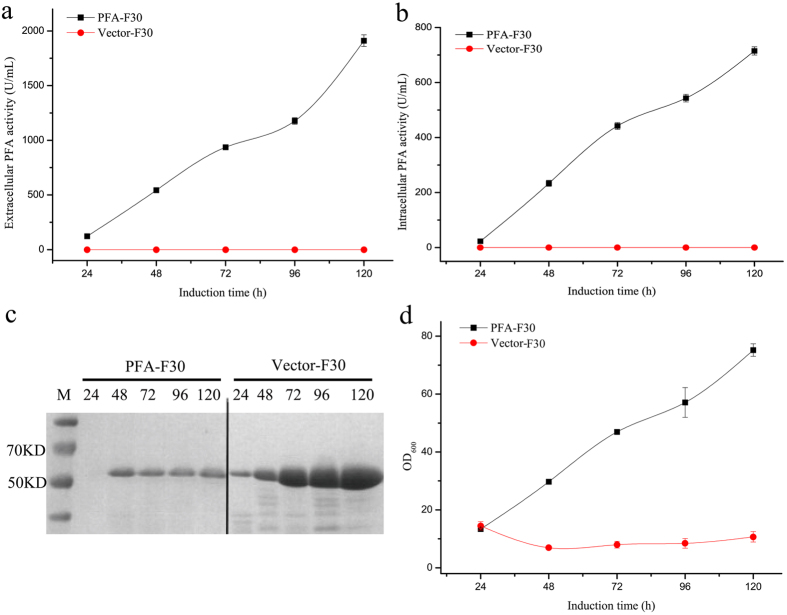
Expression of recombinant PFA in *B*. *amyloliquefaciens* in shaking-flask culture. The selected PFA-F30 (black) was cultured in a shaking flask to measure PFA expression and secretion. Meanwhile, the strain Vector-F30 (red) was used as a control. Transformants were cultured in fermentation medium for the indicated times (x axis), and PFA activity was determined using standard enzyme assays (y axis). (**a**) Extracellular PFA activity of the recombinant *B*. *amyloliquefaciens* clones; (**b**) Intracellular PFA activity of the recombinant *B*. *amyloliquefaciens* clones; (**c**) SDS-PAGE analysis of culture supernatants; (**d**) Growth kinetics of the recombinant *B*. *amyloliquefaciens* clones. M, standard protein marker; lanes 24–120, culture supernatants for 24, 48, 72, 96, and 120 h, respectively. Samples of PFA-F30 (left); samples of Vector-F30 (right). Enzyme activity is expressed as the mean of three samples, and error bars indicate standard deviation (SD).

To measure PFA expression and secretion, PFA-F30 cells were first cultured in a shaking flask using the medium and cultivation conditions that mimicked those for producing BAA. During the 5-day induction period, the maximum secretion yield was detected at 120 h, at which time the supernatant from PFA-F30 culture exhibited extracellular PFA activity of 2000 U/mL (Fig. 2a), the PFA-F30 cells exhibited intracellular PFA activity of 715 U/mL (Fig. 2b) and the Vector-F30 control (F30 harbouring empty vector pUBC19) exhibited no PFA activity.

The theoretical molecular weights of PFA and AmyA were 52 and 54 kD, respectively. The supernatant proteins were analysed by SDS-PAGE (Fig. 2c), and PFA-F30 and Vector-F30 showed a single protein band at a similar position at ~50 kD. The protein samples of PFA-F30 and Vector-F30 at 120 h were subjected to LC-MS/MS analysis. The results revealed that Vector-F30 only produced AmyA, whereas PFA-F30 produced a small amount of PFA at the same time.

During 120 h of induction, the PFA-F30 and Vector-F30 strains exhibited distinct growth rates. As shown in Fig. 2d, the growth profile of Vector-F30 entered into the stationary phase at 24 h while that of PFA-F30 showed no stationary phase, even at 120 h. The PFA-F30 strain exhibited ~7-fold higher growth than the Vector-F30 strain, as determined by final OD levels. However, SDS-PAGE analysis revealed that the secreted protein of PFA-F30 was drastically lower than that secreted by Vector-F30 (Fig. 2c). Similar results were obtained when using F31 or F32 as the host (data not shown).

These data indicate that the expression of PFA in F30 resulted in lower amounts of secreted AmyA. To our surprise, the secretory expression of AmyA located on the chromosome remained dominant in the PFA-F30 strain. Therefore, we knocked out the chromosomal *amyA* gene to investigate whether PFA yield could be enhanced in the PFA-F30 strain in the absence of a competitor for the expression machinery during starch induction.

### Deletion of gene *amyA* using the vector pNZT1-Phe in *B*. *amyloliquefaciens* F30

**Figure 3:**
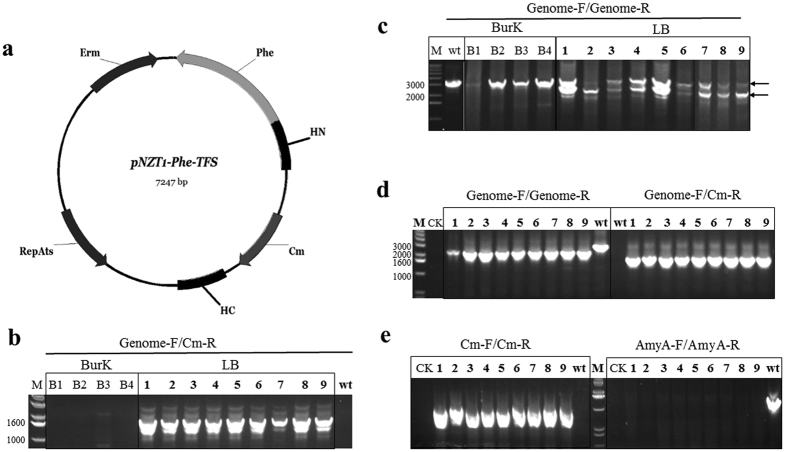
Deletion of *amyA* using the vector pNZT1-Phe in *B*. *amyloliquefaciens* F30. Bacterial colonies B1–B4, were selected from the Burk-p-Cl-Phe plate; Bacterial colonies 1–9 were selected from the LB-p-Cl-Phe plate. (**a**) Schematic representation of knock-out plasmid pNZT1-Phe-TFS; (**b**) PCR analysis with the primers Genome-F and Cm-R. The former was located on genomic DNA and the latter was located on the knock-out plasmid; (**c**) PCR analysis with the primers Genome-F and Genome-R. The arrows indicate two putive bands; (**d**) After separated by streaking, the corresponding clones were subjected to PCR analysis using the primers Genome-F and Cm-R, Genome-F and Genome-R. (**e**) Identification of the deletion gene *amyA* with the primers Cm-F and Cm-R, AmyA-F and AmyA-R. M, standard protein marker; CK, using water as the PCR template; wt, using F30 chromosomal DNA as the PCR template.

To delete chromosomal *amyA*, which was competitively expressed during starch induction, the delivery plasmid pNZT1-Phe-TFS was constructed (Fig. 3a) and introduced into F30 by electroporation. The two-step replacement recombination procedure was performed as described in the Materials and Methods section, and the pheS* gene was used as a negative-selection marker in the presence of p-Cl-Phe. According to a previous study^21^, this procedure should result in two kinds of cells: wild-type cells with the *amyA* gene on the chromosome and mutant cells with the *chloramphenicol* (*cm*) gene on the chromosome.

Equal dilution volumes of bacterial liquid were plated on LB and Burk solid media containing 0.2 M p-Cl-Phe. Hundreds of bacterial colonies were observed on the LB plate after 16–18 h of growth, while only four bacterial colonies were observed on the Burk plate after 36 h. Nine bacterial colonies randomly selected from the LB plate (named 1–9) and four bacterial colonies from the Burk plate (named B1–B4) were subjected to PCR analysis using the primers pNZT-F and pNZT-R to confirm that the vector had been removed from the strain and chromosome. The primers Genome-F and Cm-R were used to indicate whether the *cm* gene was integrated into the correct position of the chromosome in the nine clones from the LB plate (Fig. 3b). However, to our surprise, the PCR analysis of *amyA* (primers amyA-F and amyA-R) demonstrated that this gene existed in all 13 bacterial colonies. Therefore, we hypothesised that the two-step replacement recombination in most clones occurred due to the stress of p-Cl-Phe rather than before the stress of p-Cl-Phe, which would resulted in that the bacterial colonies growing on the LB-p-Cl-Phe plate were hybrids, containing two kinds of cells for two types of replacement recombinations. This was verified using the genome-F and genome-R primers, which showed that the in-frame deletion yielded a 2.3 kb amplicon, the wild-type yielded a 3 kb amplicon and the hybrid bacterial colonies yielded both 2.3 kb and 3 kb amplicons. As shown in Fig. 3c, the negative bacterial colonies B1-B4 yielded a 3 kb amplicon, while the positive bacterial colonies 1–9 yielded both 2.3 kb and 3 kb amplicons. Subsequently, bacterial colonies 1–9 were streaked on LB-starch plates. The results indicated that there were two types of clones: one capable of degrading starch, and one that could not. The latter was our aim, which was found in 10–90% of the bacterial colonies. Such bacterial colony yielded a single 2.3 kb amplicon (Fig. 3d). Subsequent PCR analyses confirmed the successful deletion of *amyA* and replacement by *cm* (Fig. 3e).

### Expression of recombinant PFA in the F30-∆amyA strain

**Figure 4:**
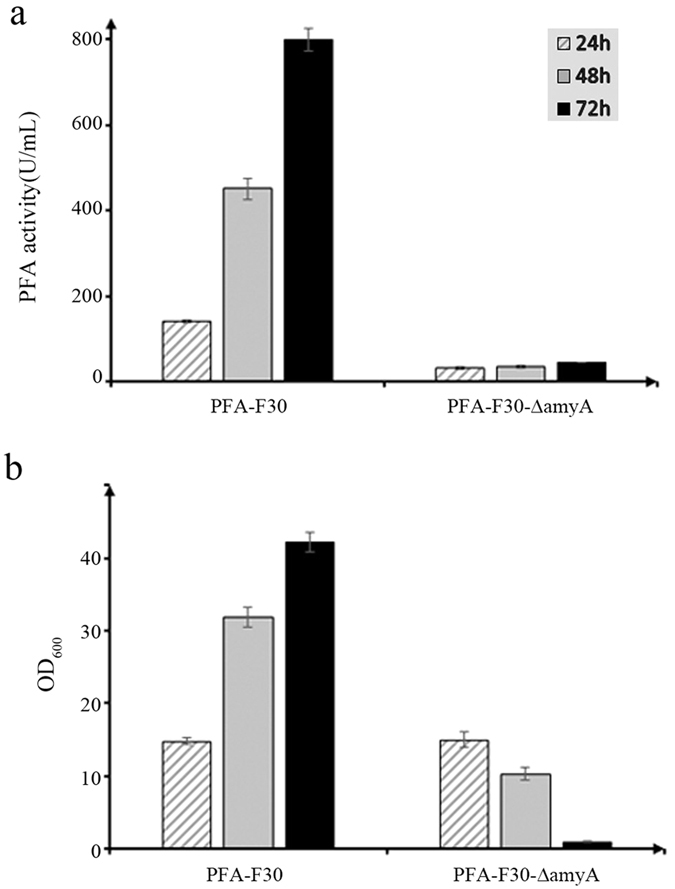
Extracellular PFA activity and cell densities of the strains PFA-F30 or PFA-F30-∆amyA. (**a**) Extracellular PFA activity of the recombinant *B*. *amyloliquefaciens* clones; (**b**) cell densities of the recombinant *B*. *amyloliquefaciens* clones. The value is expressed as the mean of three samples, and error bars indicate standard deviation (SD).

The plasmid pUBC19-PamyA-*mpfa* was used to transform the F30-∆amyA strain, generating the transformant PFA-F30-∆amyA. To evaluate the effects of the deletion, the selected transformant and PFA-F30 clones were simultaneously cultured in shaking flasks to compare the production of PFA. During the planned 5-day induction, the medium of PFA-F30-∆amyA had become too sticky to continue the cultivation for ~72 h. At that moment PFA-F30-∆amyA culture exhibited PFA activity of ~45 U/mL, which was 17 times lower than that in PFA-F30 culture (Fig. 4a). Meanwhile, the cell densities of PFA-F30-∆amyA declined gradually while that of PFA-F30 increased (Fig. 4b).

### Enzymatic properties of recombinant PFA

**Figure 5:**
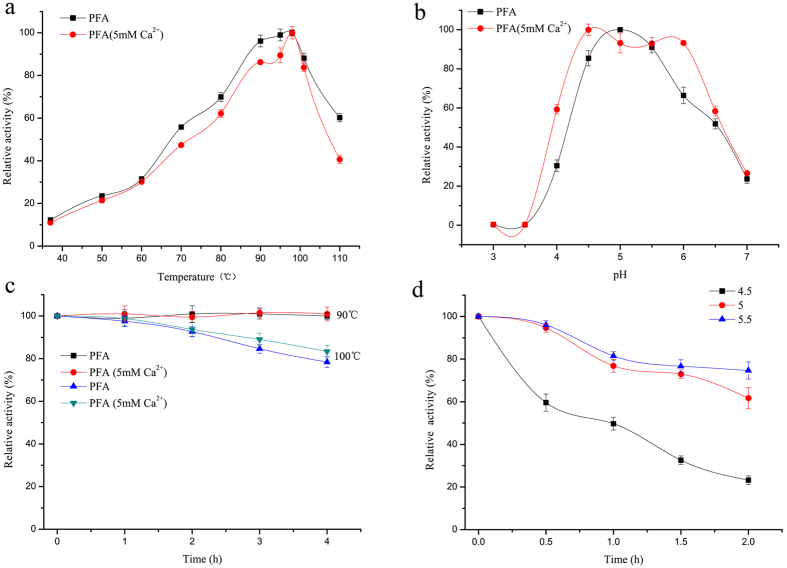
Effects of temperature and pH on amylase activity of recombinant PFA. (**a**) Temperature profile with no Ca^2+^ addition (black) or 5 mM Ca^2+^ addition (red); (**b**) pH profiles with no Ca^2+^ addition (black) or 5 mM Ca^2+^ addition (red); (**c**) stability of recombinant PFA at 90, 100 °C with no Ca^2+^ addition (black, blue) or 5 mM Ca^2+^ addition (red, green); (**d**) stability of recombinant PFA at pH 4.5 (black), 5.0 (red) or 5.5 (blue) in boiling water bath, with no Ca^2+^ addition.

The amylase activity of recombinant PFA was independent of Ca^2+^ levels. The optimum temperature for enzymatic activity was close to 100 °C with or without Ca^2+^ (Fig. 5a). However, recombinant PFA showed an optimal activity at pH 5 without Ca^2+^ and at pH 4.5–6.0 with 5 mM Ca^2+^ (Fig. 5b). As depicted in Fig. 5c, incubation at 90 °C for 4 h caused no decrease in enzymatic activity, and >80% of the enzyme activity was retained after treatment at 100 °C for 4 h. The presence of Ca^2+^ had no effect on its thermal stability. At 100 °C, ~50%, 80% and 83% of the enzymatic activity was retained after 1 h at pH 4.5, 5.0 and 5.5, respectively (Fig. 5d). Such stability properties of PFA make this enzyme a potential candidate for industrial application.

## Discussion

Compared to *B*. *licheniformis* and *B*. *subtilis*, *B*. *amyloliquefaciens* shows great potential as a host. However, unlike studies involving *B*. *licheniformis*^26^,^27^ and *B*. *subtilis*^28^, few studies have described the expression of foreign protein in *B*. *amyloliquefaciens*. Most studies have focused on the use of wild-type *B*. *amyloliquefaciens* in industrial production^29^ or strain improvement^30^,^31^. In this study, PFA was used as a model to examine the *B*. *amyloliquefaciens* protein expression system, including the requirements for transformation and gene optimisation; promoter-, vector-, strain- and genetic-specific modifications; and culture conditions. The production of PFA in PFA-F30 proved the potential of this expression system.

PFA shows great potential as a candidate for industrial application due to its thermostability, long half-life and optimal activity at low pH; however, it is difficult to express in typical protein expression systems. Several attempted strategies have failed to improve PFA yield, including optimisation of the codon promoter, signal peptide, vector and host^16^,^17^. This study employed a BAA protein expression pattern-mimicking strategy to express PFA in *B*. *amyloliquefaciens*, in which the expression system of PFA mimicked that of BAA, including the gene, the mRNA structure, vector, host and cultivation conditions. This design proved successful as assessed by comparing the various genes (*mpfa* and *opfa*), promoters (PamyA and P43) and strains (F30, F31, F32, Vector-F30 and F30-∆amyA). After optimisation, PFA-F30 was selected to measure PFA expression and secretion. During the 5-day induction period in the shaking flask, the PFA-F30 culture media exhibited a maximum PFA activity of 2000 U/mL at 120 h, while PFA-F30 cells exhibited PFA activity of 714 U/mL. Due to the thermostability of PFA, high-temperature sterilisation can be adopted after fermentation in industrial production, which could both simplify the downstream protein-purification process and release most of the intracellular PFA. Therefore, the final production of PFA reached ~2714 U/mL, about 3000- and 14-fold that of *B*. *subtilis*^18^ or *E*. *coli*^17^, respectively.

In this study, a new plasmid pNZT1-Phe, derived from the plasmid pNZT1^21^,^32^, was used to delete the gene *amyA* on the chromosome. We adopted the two-step replacement recombinant process as described previously^21^,^32^. However, the bacterial colonies on the plate were hybrids, containing two kinds of cells because of two types of replacement recombinations. This possibility was not mentioned in previous studies^21^,^32^. The Em^s^ (erythromycin sensitive) screening method described previously^21^ was also tested. All of our 13 bacterial colonies were sensitive to erythromycin, but the deletion did not happen in the bacterial colonies B1-B4. In the reference, the frequency of the second single crossover and plasmid excision has been reported to range from 1% to 99% using the plasmid pNZT1^21^. However, in this study, due to efficient negative selection in the presence of p-Cl-Phe, it was found the frequency of the second single crossover and plasmid excision was almost 100% when using the plasmid pNZT1-Phe. Therefore, the plasmid pNZT1-Phe is more efficient than pNZT1 and the method used in this study is more reasonable. Besides, although a *cm* gene was introduced into the vector pNZT1-Phe-TFS, it was not used to screen during the deletion process. Therefore, the plasmid pNZT1-Phe can be used as a simple method to introduce marker-free genetic modifications into the chromosome of *B*. *amyloliquefaciens* strains. The deletion of *amyA* on the chromosome resulted in decreased production of PFA, which supported our hypothesis that the expression and secretion of BAA in the host can help that of PFA in the same host. Because PFA is thermophilic amylase, and most amylase activity of PFA was lost at 37 °C^33^. In contrast, BAA (here also named AmyA) is mesophilic amylase, and most amylase activity of BAA was retained at 37 °C^33^. Therefore, at the culture temperature of 37 °C, PFA-F30 could not effectively utilize raw starch, the main carbon resource in the fermentation medium, without the help of BAA at the beginning until PFA in the medium accumulated to a certain amount, which may be the reason for the gradual decline in cell density in PFA-F30-∆amyA cultures and the sticky medium due to starvation. What is more, the product of starch degradation was the main inducer of the promoter PamyA but starch did not.

During starch induction, the PFA-F30 strain exhibited 7-fold higher OD levels than the Vector-F30 strain. In contrast, SDS-PAGE analysis showed that the secreted levels of AmyA from PFA-F30 were drastically lower than those from Vector-F30. It seemed that the cell density of Vector-F30 strain stopped to increase when the strain started to synthesize AmyA in large amounts. Meanwhile, synthesizing proteins in large amounts meant that a lot of ribosomes were occupied. In contrast, PFA-F30 did not occupy so many ribosomes that the strain could have unoccupied ribosomes to synthesize the proteins that was used for bacteria proliferation. Besides, these results also indicated that the competitive expression of PFA resulted in lower production of AmyA and larger cell densities. We hypothesized that this was related to the translation of recombinant PFA. However, the comparison between *mpfa* and *opfa* indicated that the translation of PFA in *B*. *amyloliquefaciens* was complicated and required further study.

In this study, although PFA was highly expressed in *B*. *amyloliquefaciens*, its production could not satisfy the demand of industry. However, there is great potential to optimise the culture conditions and properties of the host, *B*. *amyloliquefaciens*. For example, this strain usually displays strong protease activity, and productivity could be improved by deleting the proteases^34^. Moreover, additional chaperones (e.g., prsA^35^,^36^, wprA) could be introduced to aid in protein folding. Deletion of the sporation gene in *B*. *amyloliquefaciens* could prevent spore formation during the stationary growth phase, prolonging the fermentation time. In addition, many archaeal proteins have excellent enzymatic properties and great potential for application. However, many of them are difficult to express, like PFA. The BAA protein expression pattern-mimicking strategy described here could provide a new direction for the heterologous expression of other archaeal proteins in bacterial expression systems, particularly in *Bacillus*.

## Materials and Methods

### Strains, media and plasmids

The bacterial strains, genes and plasmids used in this study are listed in Table 1. *E*. *coli* and *B. amyloliquefaciens* were cultured aerobically in Luria–Bertani (LB) liquid medium or on LB agar plates (15 g agar/L LB media) at 37 °C.

When required, ampicillin (amp; 100 μg/mL for *E*. *coli*), spectinomycin (spc; 100 μg/mL for *E*. *coli*), chloramphenicol (cm; 10 μg/mL for *Bacillus*), kanamycin (km; 10 μg/mL for *Bacillus*), or erythromycin (em; 300 μg/mL for *E*. *coli* or 5 μg/mL for *Bacillus*) were added to the media.

### Design of two synthetic *pfa* genes

The GenBank accession number of *pfa* is U96622. Based on the coding sequence, we designed two synthetic *pfa* genes that encoded the wild-type PFA enzyme: *mpfa* and *opfa*.

*mpfa* was designed to mimic the codon usage and mRNA structure of BAA. To mimic the codon usage of BAA, the rare codons were substituted according to the codon usage data of *B*. *amyloliquefaciens* DSM7. In this step, the Biosoft visual gene developer (VGD) was used to perform seven rounds of calculations^37^. The calculations were performed 10 times in the first five rounds, 1000 times in the sixth round, and 2000 times in the seventh round. In the first six rounds, the sequence with the highest CAI index was chosen to carry out the next round of calculations. To mimic the mRNA structure of BAA, each sequence was examined in the seventh round according to the mRNA structure and mRNA Gibbs energy. Finally, the *mpfa* sequence was chosen and synthesised by GenScript (Nanjing, China).

*opfa* was optimised and synthesised by GenScript. The sequence was optimised using GenScript’s proprietary algorithms that replace rare codons according to the codon usage data of *B*. *amyloliquefaciens* DSM7; additional aspects, such as mRNA structure, may have been optimised.

### Construction of expression vectors

To mimic the protein expression pattern of BAA, we constructed the recombinant plasmids pUBC19-PamyA-*mpfa* and pUBC19-PamyA-*opfa* (Fig. 1a). The promoter sequence and signal peptide sequence (SP) were identical to those of *amyA* (the BAA gene of F30), and were amplified from *B. amyloliquefaciens* F30 using the primers PamyA-F and PamyA-R (Supplementary Table S1). The *mpfa* or *opfa* genes included a *Xho* I site at the N-terminal and *Pst* I site at the C-terminal, and then these fragments were inserted into pUBC19, which was constituted by the replication of pUB110^38^.

The vectors pUBC19-P43-*mpfa* (Table 1) were constructed in a similar way, except that SP was fused to the P43 promoter by overlap PCR at the beginning. All primers used in this study are listed in Supplementary Table S1.

All ligation junctions in recombinant vectors were confirmed by DNA sequencing (State Key Laboratory for Crop Genetic Improvement, Chinese Academy of Agricultural Sciences, Beijing, China).

### Preparation of *B*. *amyloliquefaciens* methylation pattern-mimicking plasmids

Strain *E. coli* B19, which contained the plasmid encoding methyltransferase genes of *B*. *amyloliquefaciens*^23^, was used to prepare the shuttle plasmids that mimicked *B. amyloliquefaciens* methylation patterns in *E*. *coli* and *Bacillus*.

Single colonies were used to inoculate LB medium. When cultures reached an optical density (OD_600_) of 0.2, arabinose was added to a final concentration of 0.2% to induce methyltransferase expression. Expression was induced overnight at 30 °C. The plasmids were extracted using a plasmid miniprep kit (Axygen).

### Preparation of *B*. *amyloliquefaciens* electrocompetent cells

Electrocompetent *B*. *amyloliquefaciens* cells were prepared as described previously^24^. In this process, two media sources were used: AM (17.5 g/L Difco^TM^ Antibiotic Medium 3; BD, USA) and NCM (17.4 g K_2_HPO_4_, 11.6 g NaCl, 5 g glucose, 5 g tryptone, 1 g yeast extract, 0.3 g trisodium citrate, 0.05 g MgSO_4_·7H_2_O, and 91.1 g sorbitol in 1 L deionised water, pH 7.2).

An overnight culture of *B*. *amyloliquefaciens* cells in AM was diluted 100-fold in 200 mL NCM medium to prepare electrocompetent cells. Cell growth was monitored by measuring the OD at 600 nm. When the OD_600_ reached 0.5, cell wall weakening was performed by adding 100 μL 100 μg/mL amp to the culture and shaking for 1 h. Next, the cell culture was cooled on ice for 20 min, and cells were collected by centrifugation at 4 °C, 4300 *g* for 5 min. After washing four times with pre-cooled ETM buffer (0.5 M sorbitol, 0.5 M mannitol, and 10% glycerol), the cells were resuspended in 2 mL ETM buffer.

### Electroporation

Plasmid DNA (1 μg) was mixed with 100 μL electrocompetent cells, and the mixture was loaded into a prechilled 1 mm gap electroporation cuvette. After a brief incubation on ice, the mixture was transformed into *B*. *amyloliquefaciens* by electroporation using the Gene Pulser system (Bio-Rad; conditions used: 2.1 kV/cm, 36 μF, and 150 Ω). The cells were diluted immediately into 1 mL recovery medium (NCM plus 0.38 M mannitol), shaken gently at 37 °C for 3 h, and then spread onto LB agar plates supplemented with the appropriate antibiotics.

### Generation and verification of the gene *amyA* deletion strain

The two-step replacement recombination procedure was performed as previously described^21^, except that we used the pheS* gene as a negative-selection marker in the presence of p-Cl-Phe in the second step^39^. In the first step, a *Bacillus* strain bearing a delivery plasmid that contained the gene replacement construct was cultivated with aeration in LB at 37 °C (a nonpermissive temperature for plasmid replication). This was done to initiate integration of the entire plasmid into the chromosome via a single crossover between the target gene and a homologous sequence on the plasmid. In the second step, a separate clone of the integrant was cultivated with aeration in LB at 30 °C for 48 h to initiate the second single-crossover event and the excision of the plasmid, which yielded p-Cl-Phe-insensitive clones with either a parental or a mutant allele on the chromosome. Colony PCR analysis was used to examine several clones for the presence of the desired mutation.

### Expression of PFA in shake-cultivation for 120 h

Bacterial colonies were used to inoculate 2 mL AM medium at 37 °C, with constant shaking at 200 rpm for 10 h. Then the bacteria were transferred into 20 mL fermentation medium (corn starch 12% [w/v], peptone 2% [w/v], corn steep liquor 0.5% [v/v], NH_4_SO_4_ 0.5% [w/v], CaCl 0.04% [w/v], K_2_HPO_4_ 0.1% [w/v], KH_2_PO_4_ 0.39% [w/v]), and induced at 37 °C with constant shaking at 250 rpm for 120 h. Samples were examined every 24 h. The culture supernatant and cells were harvested by centrifugation (12,000 *g*, 3 min, 4 °C) to analyse PFA activity, and then the cells were heated at 85 °C for 15 min to release the intracellular PFA. The PFA activity in the supernatant and cells was determined using an enzyme assay as described previously^17^. The clones harbouring the plasmid pUBC19-P43-*mpfa* or pUBC19-P16-*mpfa* were cultured in super-rich medium^38^ with km (10 μg/mL).

### Enzyme assays

PFA activity was determined by measuring the amount of reducing sugar released during enzymatic hydrolysis of 1% soluble starch in buffer NaH_2_PO_4_-citric acid at 98 °C for 10 min according to Bernfeld^40^. A control without enzyme was used. The amount of reducing sugar was measured using a dinitrosalicylic acid method as described previously^17^. One unit of amylase activity was defined as the amount of enzyme that released 1 mmol reducing sugar as glucose per minute under the assay conditions.

The activity- and temperature-dependent experiments in the temperature range of 37–90 °C were carried out in a water bath, whereas experiments in the temperature range of 90–100 °C were performed in a glycerol bath. To determine pH optimum activity, 50 mM NaH_2_PO_4_ citric acid buffer was used for pH values in the range of 3.5–7.0. Thermal stability experiments were performed at the indicated temperatures in the above-mentioned baths, and the residual activity was determined in a typical enzyme assay solution. Acid stability experiments were performed at the pH 4.5, 5.0 and 5.5 in a boiling water bath, and the residual activity was determined in a typical enzyme assay solution.

## Additional Information

**How to cite this article**: Wang, P. *et al.* A new strategy to express the extracellular a-amylase from *Pyrococcus furiosus* in *Bacillus amyloliquefaciens*. *Sci. Rep.* **6**, 22229; doi: 10.1038/srep22229 (2016).

## Supplementary Material

Supplementary Information

## Figures and Tables

**Table 1:** Strains, genes and plasmids used in this study.

Strains or plasmids	Relevant characteristics	Source or reference
Strains		
*E. coli* B19	*E*. *coli* EC135 strain harbouring pM.Bam. Used for mimicking the DNA methylation patterns of *B*. *amyloliquefaciens* and recombinant plasmid amplification.	Institute of Microbiology, CAS^23^
*B.* amyloliquefaciens F30	ACCC19885. *B*. *amyloliquefaciens* strain for producing α-amylase BAA	ACCC
*B*. *amyloliquefaciens* F31	CICC 20077. Strain for producing α-amylase BAA. A variant derived from BF7658, named BF7658-209.	CICC
*B*. *amyloliquefaciens F32*	CICC20633. Strain for producing α-amylase.	CICC
PFA-F30	F30 strain harbouring pUBC19-PamyA-*mpfa*	This study
Vector-F30	F30 strain harbouring the empty vector pUBC19	This study
F30-∆amyA	F30 derivative, Δ *amyA*	This study
PFA-F30-∆amyA	F30-∆amyA strain harbouring pUBC19-PamyA-*mpfa*	This study
Plasmids		
pUBC19	*E. coli–Bacillus* shuttle plasmid, km^R^.	Nanjing Agricultural University
pUBC19-PamyA-*mpfa*	pUBC19 with *mpfa* under the promoter and signal peptide of *amyA*, amplified from the F30 chromosomal DNA.	This study
pUBC19-PamyA-*opfa*	Replaced *mpfa* of pUBC19-PamyA-*mpfa with opfa*	This study
pUBC19-P43-*mpfa*	Replaced PamyA promoter of pUBC19-PamyA-*mpfa* with P43, amplified from pHT43	This study
pNZT1-Phe	*E. coli–Bacillus* shuttle plasmid, rolling circle replicative, Em^R^. Delivery vector pNZT1, derived from pKS1, with the pheS* gene as a negative-selection marker in the presence of p-Cl-Phe.	Nanjing Agricultural University
pNZT1-Phe-TFS	pNZT1-Phe with TFS. TFS fragment containing 1.2 kb of the Cm (0.6 kb coding region, 0.4 kb upstream of the gene and 0.2 kb downstream of the gene) amplified from pHT43, N-terminal homologous arm (HN, 0.5 kb upstream of the gene *amyA*) and C-terminal homologous arm (HC, 0.5 kb downstream of the gene *amyA*), both amplified from the F30 chromosomal DNA.	This study
