# Supplementary material for: A new strategy to express the extracellular α-amylase from Pyrococcus furiosus in Bacillus amyloliquefaciens
Source: Sci Rep. 2016 Feb 26;6:22229. doi: 10.1038/srep22229 (PMC4768087; doi:10.1038/srep22229)

A new strategy to express the extracellular α-amylase from *Pyrococcus furiosus* in *Bacillus amyloliquefaciens*

Ping Wang[§], Peili Wang[§], Jian Tian, Xiaoxia Yu, Meihui Chang, Xiaoyu Chu, Ningfeng Wu*

Biotechnology Research Institute, Chinese Academy of Agricultural Sciences, Beijing 100081, China

*Corresponding author: Ningfeng Wu, E-mail: wuningfeng@caas.cn, Tel.: +86 10 82109844;

[§]These authors contributed equally to this work.

Supplementary Table S1. Primers

Primer	Sequence (5′–3′)
PamyA-F	TCTAGAGATCTCAAGCCCGCTTTTTT
PamyA-R	CTCGAGGCCATTTACGGCTGATGTTT
pUBC19-CX-up	GGTACAAGAAAAACGAGGAAAGATGC
pUBC19-CX-down	TCGTATGTTGTGTGGAATTGTGAGC
SP-F	ATGAGAGGGAGAGGAAACATGA
SP-R	CTCGAGGGCTGATGTTTTTGTAATCGGCAAAC
P43-F	TCTAGATGATAGGTGGTATGTTTTCG
P43-R	TCCTCTCCCTCTCATGTGTACATTCCTCTCTTACC
P16-F	TCTAGACCGATAATTCCAATTTTCAT
P16-R	CTCGAG TCCTCCACCTCCCATATCTC
HN-F	GTCGACAAACTTGAGGCGATCACATC
HN-R	GAATTCTTTCAATTCTCCGGGGAATC
Cm-F	GAATTCCTAGGAGCTGGCGTAATAGCGAAG
Cm-R	GAGCTCCTGCAGGTGAGCGAGGAAGCGGAAGA
HC-F	GAGCTCGGTAATAAAAAAACACCTCCAAGCTG
HC-R	GGTACCATAAGAGAGGAGCTTGTTGAAG
pNZT-F	AGCTCCTCTCTTATGGTACCCAATTCGCCCTATAGTGAGT
pNZT-R	ATCGCCTCAAGTTTGTCGACATTTTTAAAGTATGTATACA
Genome-F	AAACTTGAGGCGATCACATCTG
Genome-R	ATAAGAGAGGAGCTTGTTGAAG
AmyA-F	ATGAGAGGGAGAGGAAACATGATTC
AmyA-R	TTATTTCTGAACATAAATGGAGACG
PFA-F	CTCGAGCATCACCACCACCATC
PFA-R	CTGCAGTTATCCAACACCACAGTAG

Underscored nucleotides indicate sites of restriction digestion.

Design of two synthetic *pfa* genes

The optimization of GenScript (*opfa*) focused on the codon optimization, involving transcription, mRNA processing and stability, translation and protein folding (http://www.genscript.com/codon_opt.html?src=gssb). According to our BAA protein expression pattern-mimicking strategy, *mpfa* was designed to mimic BAA as many aspects as possible, which included the mRNA structure of BAA in addition to codon optimization. As shown in Figure S1, the mRNA structure of *opfa* was different with that of BAA while the mRNA structure of *mpfa* was similar with that of BAA.

Figure S1 the mRNA structures of *BAA*, *opfa* and *mpfa*. All the mRNA structure were calculated on the website (http://rna.tbi.univie.ac.at/cgi-bin/RNAfold.cgi)."

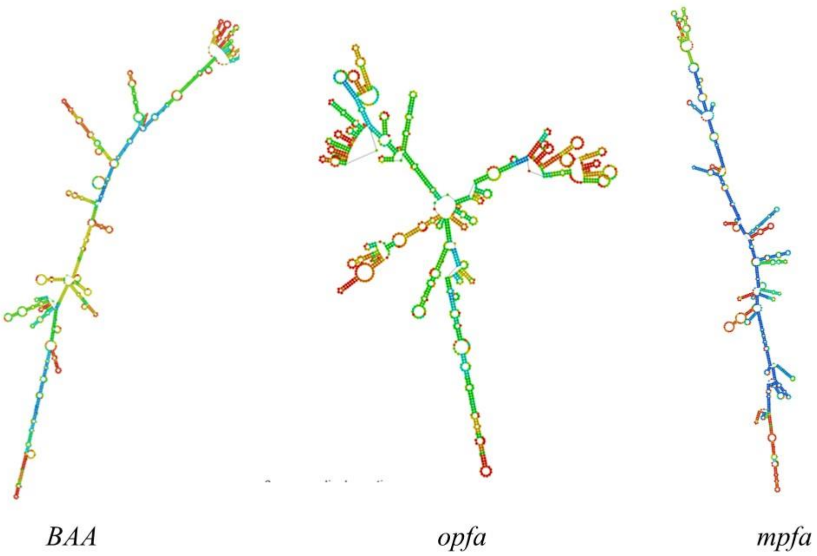

Supplement: Supplementary Information [file srep22229-s1.pdf]
